# Intensifying poultry production systems and the emergence of avian influenza in China: a ‘One Health/Ecohealth’ epitome

**DOI:** 10.1186/s13690-017-0218-4

**Published:** 2017-11-27

**Authors:** Marius Gilbert, Xiangming Xiao, Timothy P. Robinson

**Affiliations:** 1 Spatial Epidemiology Lab (SpELL), Université Libre de Bruxelles, CP264/03, 50, av FD Roosevelt, B-1050 Brussels, Belgium; 2 Fonds National de la Recherche Scientifique (FNRS), Brussels, Belgium; 3 Department of Microbiology and Plant Biology, Center for Spatial Analysis, University of Oklahoma, Norman, OK USA; 4 Institute of Biodiversity Science, Fudan University, Shanghai, 200433 China; 5 Policies, Institutions and Livelihoods (PIL), International Livestock Research Institute (ILRI), Nairobi, Kenya; 6 Livestock Information, Sector Analysis and Policy Branch (AGAL), Food and Agriculture Organisation of the United Nations (FAO), Viale delle Terme di Caracalla, 00153 Rome, Italy

**Keywords:** Avian influenza, Zoonoses, Global health, Ecosystem health

## Abstract

Several kinds of pressure can lead to the emergence of infectious diseases. In the case of zoonoses emerging from livestock, one of the most significant changes that has taken place since the mid twentieth century is what has been termed the “livestock revolution”, whereby the stock of food animals, their productivity and their trade has increased rapidly to feed rising and increasingly wealthy and urbanized populations. Further increases are projected in the future in low and middle-income countries. Using avian influenza as an example, we discuss how the emergence of avian influenza H5N1 and H7N9 in China was linked to rapid intensification of the poultry sector taking place in landscapes rich in wetland agriculture and wild waterfowls habitats, providing an extensive interface with the wild reservoir of avian influenza viruses. Trade networks and live-poultry markets further exacerbated the spread and persistence of avian influenza as well as human exposure. However, as the history of emergence of highly pathogenic avian influenza (HPAI) demonstrates in high-income countries such as the USA, Canada, Australia, the United Kingdom or the Netherlands, this is by no way specific to low and middle-income countries. Many HPAI emergence events took place in countries with generally good biosecurity standards, and the majority of these in regions hosting intensive poultry production systems. Emerging zoonoses are only one of a number of externalities of intensive livestock production systems, alongside antimicrobial consumption, disruption of nutrient cycles and greenhouse gases emissions, with direct or indirect impacts on human health. In parallel, livestock production is essential to nutrition and livelihoods in many low-income countries. Deindustrialization of the most intensive production systems in high-income countries and sustainable intensifications in low-income countries may converge to a situation where the nutritional and livelihood benefits of livestock production would be less overshadowed by its negative impacts on human an ecosystem health.

## Background

Many factors that can influence the transmission of infectious diseases are changing rapidly over time and this results in new patterns of disease emergence and spread [1]. More specifically, in the last few decades, the emergences of several zoonoses such as avian influenza (AI) H5N1 or H7N9, the middle east respiratory syndrome (MERS) in the Arabic peninsula, Q-fever in the Netherland or Ebola in Western Africa have each time been considered as unprecedented events. We understand some of the reasons for these emergence events retrospectively, but we fail to predict them adequately. These emergences of zoonoses are of particular human health concerns. They caused several hundred human infections with high fatality rates and AI and MERS, for example, could gain the capacity to transmit between humans, and to cause epidemics of unknown magnitude and impact. We argue that the failure to predict these emergences may be due to two main reasons. First, predictions are most often based on what is currently known of a disease and its risk factors where it circulates, but we fail to consider factors that could be important in different areas or under different conditions. Second, gradual changes in anthropogenic, environmental and wildlife factors are difficult to monitor, and the result of their potential interactions through different conditional feedback loops are inherently difficult to predict. Recognizing these challenges, the FAO publication “World Livestock 2013: changing disease landscapes” [2] proposed to structure the understanding and mitigation of emerging zoonoses by considering the *pressure*, *state* and *response* framework used in environmental sciences. The description and understanding of *pressures* is somewhat larger than the classical focus on risk factors, as it entails looking at broad-scale spatio-temporal pattern of changes in generic anthropogenic, environmental and wildlife drivers of change. For example, the description of changes in animal trade networks in response to new socio-economic conditions may influence a broader set of diseases that can transmit through those trade networks. Similarly, political and socio-economic instability and migration crises have disruptive implications for many human and animal diseases alongside other environmental and wildlife factors. Studying the *state* strives to understand how changes in *pressures* have resulted in disease outcomes, or may influence disease outcome in the future. It is disease-specific and aims towards a fine understanding of the mechanisms by which changes in those anthropogenic, environmental and wildlife drivers may influence the emergence, spread or persistence of a particular disease. Finally, the *response* looks at the different options of intervention at the *pressure* or *state* level to prevent emerging zoonoses or to mitigate their impact [2]. In this paper, we discuss different sets of *pressures* that can be linked to emerging zoonoses, taking avian influenza as an example. *Pressures* typically include anthropogenic (trade of live animals and animal products, distribution of farms and livestock, farming practices, farmers’ behavior, product price and farmer’s income, hunting practices, game animal transport, short-term mobility and migration of populations, socio-economic instabilities, state of veterinary services, regulation), environmental (climatic variables, land-use, land-cover, habitat connectivity) and wild-host related drivers (wildlife or vector distribution and population dynamics, vector capacity, reservoir capacity).

## Main text

**Fig. 1:**
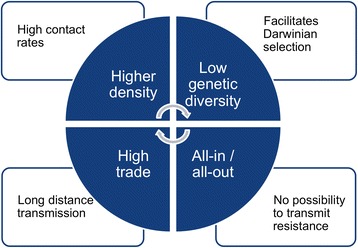
Characteristics of intensifying livestock production systems and their consequence on the spread and evolution of emerging infectious diseases

When it comes to infectious diseases, key elements of the *pressures* are the changing demographics of host populations and of their connectivity because these will have a strong influence on the short-range and long-range potential transmission dynamics. For livestock diseases, an important element of the *pressures* is therefore the level of intensification as it can directly influence the transmission and evolution of diseases through several mechanisms (Fig. 1). Intensification refers to the various processes by which livestock production and trade systems can improve the overall outputs (typically the quantity of meat, eggs, or milk produced) per unit of input (typically the number of animals) [3, 4]. Intensification usually entails increases in animal numbers and densities, the use of specialized breeds and specific feed to increase conversion ratios, faster production cycles, synchronous all-in/all-out production (i.e. raising batches of young animals of batches of homogeneous ages and harvesting them at the same time), and this is usually linked to long-distance trade through complex value chains involving several intermediates between the producers and the consumers. Each of these changes may potentially change transmission patterns and the evolutionary conditions of prevailing pathogens (Fig. 1). Higher densities results in higher contact rates between individuals, reduces the cost of virulence, favouring more virulent pathogens [5]. When contact rates are particularly high, a highly virulent pathogen may indeed be better able to transmit before it kills its host, compared to a situation with low contact rates that would select for milder pathogens. In addition, the low genetic diversity of specialized breeds may further facilitate the Darwinian selection of specialized pathogens. All-in/all-out practices prevent the maintenance of natural resistance gene in host populations compared to more extensive settings where individuals who may have survived a local outbreaks would be used to restock, and mathematical models indicate that this may have implication on the evolution of virulence and host resistance [6]. Finally, longer value-chains along trade networks give more opportunities for long-distance transmission of any emerging pathogen. Therefore, when all these factors are combined, intensification of animal production results in high risk of disease emergence that can only be prevented through careful disease prevention and control measures. With this in mind, what is the current state of these dynamics in areas important for avian influenza emergences?

At the global scale, changes in global production of several livestock commodities, called animal-sourced food (ASF) such as milk, eggs, poultry and pig meat have been particularly marked in the last 50 years. But these changes have been taking place a lot faster in low and middle-income countries (LMICs) than they were in high-income countries (HICs). For example, according to FAOSTAT, between 1975 and 2013, pork production was multiplied by 5.76 and 1.50 in LMICs and HICs, respectively [7]. Whilst high-income countries represented the largest share of production up until the 90s, fast rises in transition economies of countries such as China or Brazil brought a shift, with LMICs now representing the largest share of production. In 1975, the production of pork meat was close to 25 million tons in HICs and only 13 million tons in LMICs. In contrast, in 2013, these figures were 37.5 and 75 million tons, respectively [7]. As a consequence, both the absolute production and growth rate of ASF are now higher in LMICS than HICs. These changes in production have resulted only partly from demographic growth. For example, Robinson et al. [8] estimated that only 11% of the demand growth for animal protein in China between 2000 and 2030 could be attributable to demographic changes, 78% could be attributable to changes in demand per capita, and 11% to the combined effects. Higher income in transition economies translate into changes in dietary preferences toward higher consumptions of animal-source food per capita, and this has driven the largest share of increases in production in LMICs so far. Interestingly, projection for future productions made by Alexandratos and Bruisma [9] only confirm these trends for the future, with LMICs becoming by far the largest producers of eggs, milk and poultry and pig meat by 2030. Increases in the global trade of live animals have also been particularly significant, especially for pigs and poultry. If one integrates the number of animals by their travelled distance, the number of pigs.km and chicken.km were multiplied by a factor of 8.33 and 3.01 respectively between 1985 and 2013 according to estimates made from FAOSTAT [7] trade matrices. Today, putting end-to-end the travelled distance of all chickens transported globally gives an estimated chicken.km distance of 13,000 astronomic units (one astronomic unit correspond to the distance between the earth and the sun, i.e. 149.6 million km, this calculation is an approximation made using great circle distance between countries capitals). Thus, both the demographics of animal hosts and their connectivity have changed drastically in the last few decades, with strong geographical differences between regions and countries. In particular, fast intensifications processes taking place in transition economies such as China or India may have strong epidemiological implications for avian influenza, other emerging zoonoses and antimicrobial resistance.

**Fig. 2:**
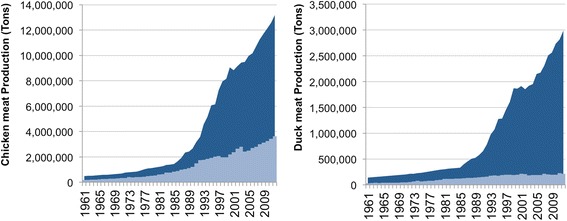
Increase in chicken (*left*) and duck (*right*) meat production in China (*dark blue*) compared to the total production of Cambodia, Indonesia, Lao, Thailand and Vietnam (*light blue*) according to FAOSTAT [7]

China occupies a particular position in the global epidemiology of avian influenza viruses, with two of the most important avian influenza viruses with pandemic potential emerging in the country. In 1996, the highly pathogenic avian influenza (HPAI) H5N1 virus emerged in southern China, and persisted locally until it started spreading internationally in 2004 [10]. By 2006, the HPAI H5N1 virus had spread across over 60 countries in Asia, Europe and Africa [11]. Still today, the H5N6 and H5N8 HPAI viruses that caused important epizootics in the USA (winter 2014/2015) and Europe (winter 2014/2015 and 2016/2017) originated in China [12], and share an H5 genes with many HPAI H5N1 that were circulating in China in the previous years, and that reassorted with other avian influenza viruses in eastern Asia. The HPAI H5N1 was able to infect human (907 cases between May 1997 and April 2015), with an overall case-fatality risk recently estimated at 53.5% among the reported infected people [13], but never evolved to sustained human-to-human transmission. An H7N9 low pathogenic avian influenza (LPAI) emerged nearby Shanghai in China in 2013 and has since caused annual peaks of reported human infection with a case-fatality risk ranging between 34 and 47% depending on the epidemic wave, among the people reporting infections in hospitals [14]. Although the last waves of infection appeared to have many more cases than the four previous ones, the virus has still not showed evidence of human-to-human sustained transmission. In the last 30 years, China massively intensified the production of poultry – both chicken and ducks (Fig. 2) - in response to fast changes in demands. These changes in demand are themselves linked to increasing urban population (who can no longer produce poultry as compared to rural populations who may keep backyard poultry for self-consumption), to the increase in per capita meat consumption (when populations get richer, then tend to change their diet toward more animal protein consumption) and to human population growth [2]. This resulted in an unprecedented upscale of production that outpaced the increase of total production in all other Southeast Asia countries (Fig. 2). HPAI H5N1 emerged in China in 1996 [10], and one can note a slow down on poultry production in the following years, before production continued increasing. The density of domestic ducks has been showed to be the main risk factor consistently found associated with the risk of persistence and spread of HPAI H5N1 in poultry [15]. This relates to the fact that domestic ducks can go through HPAI H5N1 infection and shed virus in the environment without showing strong clinical signs of diseases [16], in contrast to chicken, and may hence be silent shedders of the virus. With that in mind, one can imagine the consequence in terms of emerging risk of the rapid scaled up duck’s production showed/observed in Fig. 2. The risk or emergence, spread and persistence of avian influenza in Asia may have been exacerbated by the characteristics of poultry trade. A large fraction of poultry products remain traded through live-poultry markets in China, Vietnam, Bangladesh and Indonesia, and theses live-poultry market can be connected over large geographical distances through trade links. In China, trade connections were found between markets located several hundred kilometres apart. Moreover, our recent results indicated that the risk of human infection by H7N9 LPAI viruses linked to markets was strongly influenced by the local density of these markets [17]. Therefore, the intensification of poultry production in China resulted in unprecedented increases in densities and connectivity of poultry populations, providing ample opportunities for farm-to-farm transmission and human exposure. One should note, however, that emerging HPAI viruses are by no mean specific to middle-income economies where one can assume lower biosecurity standards at the farm level. In fact, novel emergences of HPAI viruses from an LPAI progenitor happened many times in high-income countries within intensive poultry production and comparatively higher biosecurity standards in the Netherlands (2003 H7N7), Italy (1997 H5N2, 1999 H7N1), United Kingdom (1959 H5N1, 1963 H7N3, 1979 H7N7, 1991 H5N1), USA (1983 H5N2), Australia (1976 H7N7, 1985 H7N7, 1992 H7N3), or Canada (1966 H5N9) [18] event at times when these countries where themselves intensifying their production. The specificity of Asia in comparison to these other countries is perhaps the importance of these live-poultry markets, which allow short and long-distance transmission between farms, and a strong exposure of consumers.

Aside from fast demographic and connectivity changes, other *pressures* may also have contributed to the emergence of avian influenza in many countries, and in particular in Asia. Wild water birds of the *Anatidae* family (ducks, geese and swans) form the main wild reservoir of avian influenza viruses, harbouring a wide diversity of types and subtypes. However, the habitat of these water birds has been under strong pressure following agriculture intensification in wetlands. A striking example is Poyang Lake in Jiangxi province. It is the largest freshwater lake in China used by 500,000 wild birds belonging to 75 species as part of their habitat, depending on the season. The lake is surrounded by croplands, which over time have gradually replaced natural wetlands with intensively cropped rice paddy fields [19]. Rice and duck farming are strongly associated in many Asian countries, and 26 million duck and geese and 21 million chickens are raised in the 10 counties surrounding Poyang lake. In addition to these duck and chicken farms, some farmers developed new activities of farming wild geese, which are allowed to fly into the lake daily before being brought back to the farm, and this sector may represent six million geese in the Poyang Lake area alone. A few years ago, a multidisciplinary study involving the GPS tracking of both domestic ducks and wild waterfowls showed that they both fed on post-harvested rice paddy fields, offering many opportunities for indirect transmission through contaminated faeces [20]. Land-use changes associated with intensification of both rice and poultry production have created vast interfaces between the domestic and wild avifauna, creating many opportunities for transmission of viruses between wild and domestic birds, and vice-versa. This is still changing. A recent study showed that a vast area of intensive cropping developed in north-eastern China in the last 10 years. This may now have formed a new ideal interface for wild and domestic poultry [19] in the northwest of South Korea, possibly creating a new important zones for avian influenza reassortment and transmission in North-eastern Asia. In summary, the emergence of avian influenza HPAI H5N1, H5N6, H5N8 and LPAI H7N9 relates to intensified poultry production systems in landscape rich in wetland agriculture and wild waterfowls habitats, with the risk of spread and persistence being exacerbated by trade networks and live-poultry markets.

Although these conditions are somewhat specific to parts of Asia, it would be wrong to consider that the processes that they reveal are equally specific. Several other recent emerging zoonoses followed decades of increases in stock, as quantified from FAOSTAT [7]: the emergence of Q-fever in the Netherlands in 2007 [21] followed a period of rapid increase in goat populations, the emergence of the Middle-East Respiratory Syndroms (MERS) in the middle-east in 2012 [22] followed decades of increases in camel numbers in the Arabian peninsula. Similarly, the recent emergence of an indigenous HPAI H5N1 (distinct from the Asia one) in France [23] followed several years of increase in duck populations. Of course, intensification of animal production is usually paired with better bio-security and investment in animal health prevention and control. However, as the emergence of HPAI viruses in numerous high-income countries demonstrates, biosecurity is far from perfect and allows these emergences to take place occasionally, with devastating consequences for the livestock sector when these diseases are not zoonotic, and with significant public health implications when they are.

The emergence of zoonoses is only one of the many challenges faced by the livestock sector in terms of sustainability and public health. Another important challenge is the question of antimicrobials uses in ASF production, either used as food additive (a practice that is increasingly forbidden), or overused as preventive or curative drug, which contributes to the increasingly important problem of antimicrobial resistance [24]. However, the role played by the livestock sector differs greatly depending on the context. In high-income countries (HICs), cheap production of ASF, mainly milk, eggs, meat, and their over-consumption by some, contributes to the obesity epidemic, plays a significant role on the global level of disrupts nutrient cycles and contributes to greenhouse gases emissions. By contrast, in low-income countries (LICs), 165 million children are stunted or live in a state of poor nutrition that could be addressed through local production and consumption of ASF, rich in energy and essential nutrients. In addition, in those most vulnerable countries, livestock play important and diverse roles for agricultural populations through the provision of manure and traction power, an alternative to bank systems, and insurance against hard times. It is estimated that livestock contributes to the livelihood and resilience of nearly 800 million poor smallholders throughout the world [8]. Depending where in the world it is located, and how it is managed or integrated, the livestock sector can thus both have very positive and very negative impacts on human and ecosystem health.

**Fig. 3:**
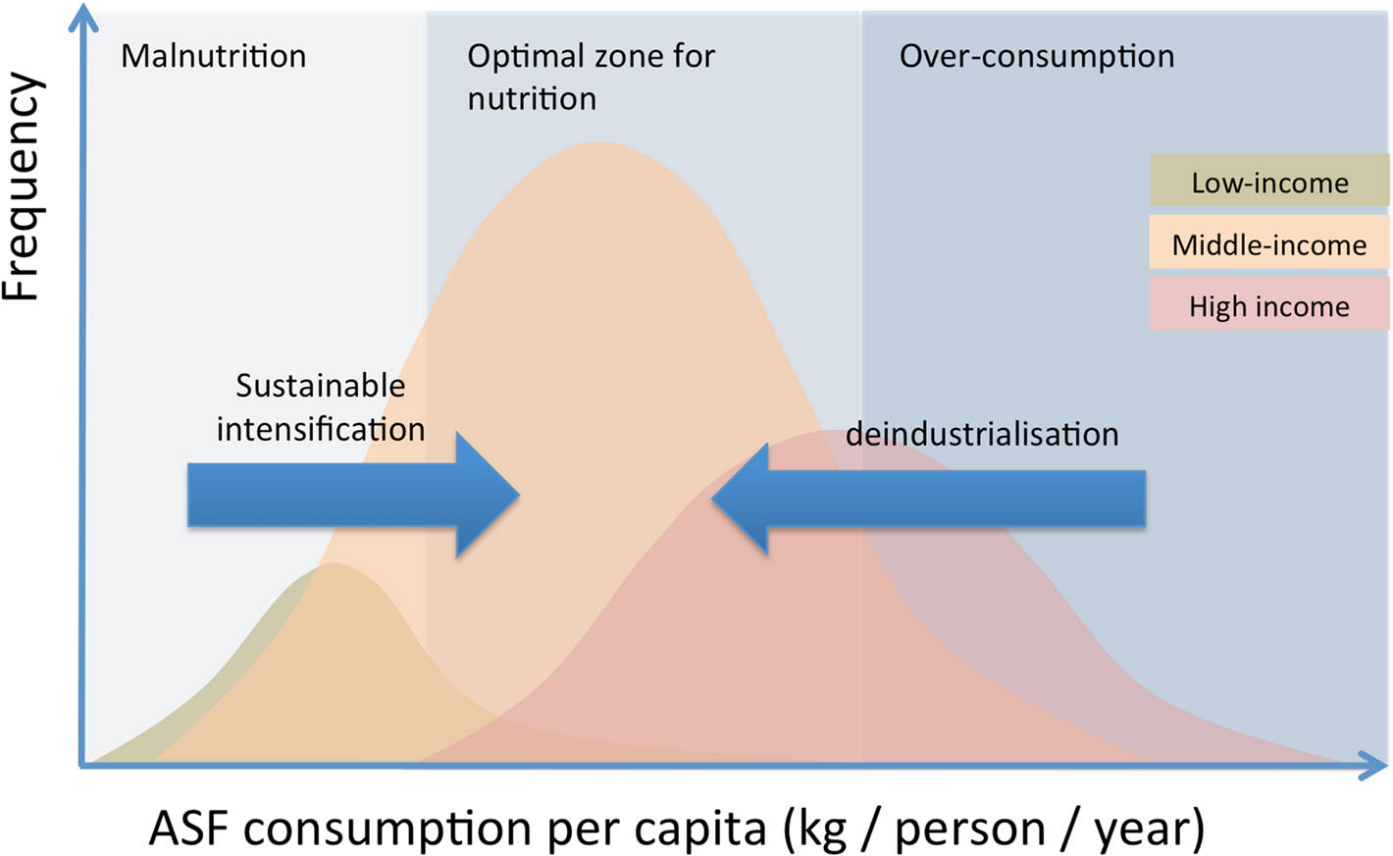
Schematic diagram illustrating the consumption of animal source-food (ASF) in low-income, middle-income and high-income countries, and suggested path toward reducing externalities while increasing societal benefits

We argue that a balanced and sustainable future for livestock production systems entails acting on both ends of the consumption and production spectrum (Fig. 3). In the poorest countries, the concept of sustainable intensification recently emerged and may help improving productivity with limited environmental and health externalities. There are many views on the concept of sustainable intensification, but we consider it here as embracing the issues of environment, pubic health and social equity: promoting equitable access to nutritious food, local nutrient recycling, the use of improved feed and other management practices to minimize greenhouse gazes emissions, and a reduction of stocking densities combined with careful biosafety practices to reduce dependence on antimicrobials and prevent the emergence of infectious diseases. In middle-income countries (MICs), where consumption patterns are already at moderate levels, improvements could be brought at the farm level in terms of good management practices, disease prevention and nutrient recycling, with the aim to increase products quality rather than quantities, and reducing the environmental impact of the current production levels. In HICs, de-industrialization of animal production would restore a more balanced connection between land and farm production, a reduction of energy inputs (e.g. fertilizers, pesticides, mechanization), better nutrient recycling through the local production of food and improved manure management, an overall reduction in stocking rates paired with an increase in products quality and prices and considerable animal welfare improvements.

## Conclusions

The emergence of avian influenza viruses is linked to intensification of the poultry sector, both in high-income countries, where evidences link de-novo HPAI emergences to intensive poultry production systems, as well as in rapidly growing economies such as China, where the intensification of chicken and duck production at the interface with the wild virus reservoir supported the emergence and maintenance of several viruses of global public health relevance, such as the H5N1 and H7N9 viruses. In the short term, better biosecurity and prevention practices, improved and more frequent cleaning and disinfection at the level of farm and live-poultry markets may contribute to reduce the circulation of the disease in poultry and the human exposure to prevailing viruses in countries sharing similar conditions. In the long run, if one consider the wider set of direct and indirect impact and benefits of animal production, one could act on both ends of the livestock production systems intensification spectrum, through deindustrialization of production in HICs and sustainable intensification in LICs, and thereby optimize the societal benefits of ASF production while reducing its main externalities on human and ecosystem health.
